# Evaluation of questionnaire as an instrument to measure the level of nutritional and weight gain knowledge in pregnant women in Poland. A pilot study

**DOI:** 10.1371/journal.pone.0227682

**Published:** 2020-01-15

**Authors:** Ewa Mierzejewska, Talita Honorato-Rzeszewicz, Dorota Świątkowska, Marzena Jurczak-Czaplicka, Tomasz Maciejewski, Anna Fijałkowska, Jagna Szulc-Kamińska, Anna Czach, Hanna Nałecz, Dorota Szostak-Węgierek, Katarzyna Szamotulska

**Affiliations:** 1 Department of Epidemiology and Biostatistics, Institute of Mother and Child, Warsaw, Poland; 2 Obstetrics and Gynecology Clinic, Institute of Mother and Child, Warsaw, Poland; 3 Department of Child and Adolescent Health, Institute of Mother and Child, Warsaw, Poland; 4 Department of Cardiology, Institute of Mother and Child, Warsaw, Poland; 5 Department of Clinical Dietetics, Warsaw Medical University, Warsaw, Poland; Aga Khan University Medical College Pakistan, PAKISTAN

## Abstract

Pregnancy is a period in life in which women are willing to improve their lifestyle. Providing proper information for these women is crucial for their health and the health of their offspring. Clear information about weak points in their nutritional and weight gain knowledge is the first step for proper health care assistance. There are a few previous studies evaluating the nutritional and weight gain knowledge of pregnant women. In the few studies available, different approaches were taken and there was no wider discussion on the content of the questionnaires attempting to measure level of knowledge. The aim of this study, designed in a pilot fashion, was to test the adequacy of the questionnaire as a research instrument in a group of 139 pregnant Polish women. The developed instrument is a 33-item questionnaire comprising four domains: weight gain, importance of nutrients, quality and quantity of food intake. The results of this study indicate that the questionnaire is stable and internal consistency is acceptable (Cronbach’s alpha > 0.7) for dimensions with more than four items. For dimensions with less than four items, internal consistency was poor (Cronbach’s alpha < 0.7). The cumulative explained variance for domains weight gain, importance of nutrients, quantity and quality of food intake was 54.74%, 42.74%, 54.42% and 48.99% respectively. Results from validity, reliability and factor analysis indicate that the questionnaire is adequate for its purpose.

## Introduction

The United Nations have declared the years of 2016–2025 the Decade of Action on Nutrition [1], in an effort to improve nutritional status globally. Inadequate diet and weight gain are important modifiable risk factors for non-communicable diseases, which are the leading cause of morbidity/mortality in the European region [2].

One group requiring special attention on diet quality, vitamin, and mineral intake are pregnant women. Maternal nutrition is a key factor for the health of the mother and for fetal development. Conditions such as preeclampsia, anemia, neural tube defects, depression and cognitive dysfunction are associated with lack of proper intake of folic acid [3,4], iron [5], calcium [6],vitamin D and iodine [7,8]. Poor nutrition during fetal development has long been demonstrated by Barker to have long term effects later in the adult life of the offspring [9].

On the contrary, maternal overnutrition also has a long-term effect on the health of the offspring and the mother herself. Women who are overweight or obese have lower fertility rates, longer trying to conceive periods, and are more likely to experience caesarean section and pregnancy loss [10–12].These women have higher chances of developing pregnancy induced hypertension, pre-eclampsia, gestational diabetes, labor and postpartum complications, and many other comorbidities [13,14].The offspring have an increased risk of diabetes, cardiovascular diseases and, metabolic syndrome during adult life [15], via fetal programming and developmental disturbances [16]. Other adverse outcomes are congenital malformation, large for gestational age, dystocia and neonatal hypoglycemia, stillbirth, and infant death [17,18].

One in every four women in Poland is overweight or obese at the beginning of pregnancy [19]. It is known from a previous European report that Poland had a slightly lower proportion of overweight and obese women (25.6%) compared to the European average (30–37%) in 2010 [20]. However the proportion of overweight and obese women of reproductive age in Poland is increasing. More recent national reports indicate that for the period of 2009–2014, proportions increased from 28.1% to 41.2% [19,21], especially among women of 20–29 years (+11.8%) and 30–39 years (+17.1%).

This observed increase in obesity/overweight in Poland seems to follow nutritional trends of higher caloric intake [22]. Usually this excessive intake is based on empty calories ,i.e., insufficient in essential nutrients and high in contents of fat, sugar and sodium [23–25]. As consequence, it has been observed that the everyday diet of pregnant women does not meet nutritional recommendations [26,27].

Nevertheless, pregnancy often motivates women to change towards a healthy nutritional behavior [28,29]. However, women’s capability to implement a healthy diet is determined not only by socioeconomic factors [30,31], but also by nutritional knowledge and familiarity with recommendations [32]. Improper knowledge or the lack of it, often as consequence of information obtained from unreliable sources [33], may result in an unhealthy diet, despite the positive attitude women might have about healthy changes. A questionnaire, assessing what is known by pregnant women can contribute to elucidate which information health care providers need to highlight.

There are many studies assessing nutritional knowledge in the general population, but very few in pregnant women. The studies in pregnant women should take into account specific recommendations such as avoidance of certain foods or additional caloric intake. The few available questionnaires are insufficient to evaluate practical knowledge about quality and quantity of food intake, i.e. what to eat and what not to eat, according to official recommendations for pregnant women. Some of the studies addressed theoretical aspects of knowledge, e.g., dietary sources of particular nutrients and their role during pregnancy or health problems related to inadequate food intake [25,31,33], which microorganisms may cause infections [25,33], details regarding supplementation of micronutrients [31,33] or unsafe foods [25,34]. Therefore, we designed a questionnaire to evaluate nutrition and weight gain knowledge in a population of Polish pregnant women. The aim of this study, designed in a pilot fashion, was to test the adequacy of the questionnaire as a research instrument to evaluate nutritional knowledge in pregnant women.

## Material and methods

### Subjects and settings

Between October and November 2017, the first 50 pregnant women from first, second and third trimester willing to participate in the study were selected. Pregnant women were recruited by 14 midwives from four outpatient clinics: three maternity hospitals in Warsaw (Institute of Mother and Child, Inflancka Hospital and Hospital of the Holy Family) and one polyclinic in Siedlce (Mother and Child Centrum). These institutions offer care for patients with normal pregnancy, with common health problems or with complex disorders, with no referral needed. The pregnant women answered the questionnaire while expecting a regular visit with the midwives or medical doctors at their respective outpatient clinic.

### Questionnaire development

The questionnaire is part of an ongoing nationwide Polish survey designed to provide an overview of the nutritional, weight gain, and physical activity status of pregnant women. The survey is divided in the following sections: nutritional and weight gain knowledge, physical activity knowledge, 3-day food record, validated Polish food frequency questionnaire (FFQ) [34] and, validated Polish version [35] of the pregnancy physical activity questionnaire (PPAQ) [36]. Additionally, the survey also collected demographic information such as age, marital and socioeconomic status, smoking habits, and alcohol consumption. Also obstetric, labor, and outcome information of the pregnancy were collected. This study explores the questionnaire regarding nutritional and weight gain knowledge during pregnancy. The questionnaire was based on national [37,38] and international nutritional recommendations for weight gain and nutrition during pregnancy [39].

Pregnant women were asked about their source of information regarding nutritional knowledge during pregnancy. Subsequently, the sources of information were classified as reliable or non-reliable. The source of information was considered reliable when it was obtained from at least medical doctors, midwives, dietitian, technical books or pre-natal education courses. Non-reliable sources of information were those obtained via friends, family, internet, magazines, radio or television.

### Validity

For the evaluation of content validity, one nutritionist and public health specialist and one dietitian examined relevance, clarity and necessity of each item. For the evaluation of face validity, nine pregnant women were selected from the obstetric ward from the Institute of Mother and Child Hospital in Warsaw between August and September 2017 to answer a pre-pilot questionnaire. Along with three midwives, the questionnaire was reviewed by the pregnant women in order to check item clarity, ambiguity and length of the questionnaire. For the evaluation of construct validity, a pilot cross sectional study with 150 participants was designed.

### Dimensions

Exploratory factor analysis (EFA) was used to determine the underlying structure of the questionnaire. Principal component analysis was performed to explore different dimensions of the questionnaire. Factors with eigenvalues >1, i.e., with sufficient explained variance, were retained to compose the domains. Oblique or orthogonal rotation was used depending on the best fit correlation between items within each domain. Bartlett’s test of sphericity was applied to test if the items were related and thus suitable for factor analysis. Kaiser-Meyer-Olkin statistics (KMO) was used to evaluate if the structure of the correlations between items allowed factor analysis.

### Score system

Each item was given a score of 100 for the correct answer and 0 for the wrong answer. A full point was awarded when the correct answer was selected from multiple choice items. For Likert scale items, a full point was given when the correct answer was selected and half point was given when the closest correct answer was selected. Nine items had subitems. For these, the score for the item is the average of the subitems’ score. The total score is the sum of all scores divided by the number of items.

In the questionnaire, some false-positive answer options were added in order to prevent the ballot effect bias, in which answers presented are overestimated and answers not presented are underestimated [40].

### Reliability

Cronbach’s alpha is a coefficient of reliability which evaluates if the test is accurately measuring the variable of interest. Cronbach’s alpha was used to obtain the reliability within the dimensions obtained from EFA.

### Item difficulty and item discrimination

For each item the factor loadings, Cronbach alpha if item deleted, item difficulty and item discrimination were calculated. Factor loading is a Pearson correlation coefficient between a given item and the factor and it was used to decide whether a certain item should be included or excluded to compose a factor. Item difficulty is the percentage of correct answers of an item. For the calculation of item discrimination (ID), i.e., how efficiently an item differentiates knowledge levels, the pregnant women were divided into low (bellow 25^th^ percentile) and high (above 75^th^ percentile) scoring groups for the total score of the questionnaire. The number of correct answers on a given item within these groups was used to calculate item discrimination [41]. Item discrimination was not calculated for items with subitems because the scores for these items were continuous.

For all analyses, a p-value <0.05 was considered statistically significant. Analyses were performed with IBM Statistical Package for Social Sciences, version 25 (Supplier: IBM Corporation. Armonk, NY, USA).

### Ethical approval

Pregnant women participating in this pilot study provided oral informed consent and permission for the use of their data for the purpose of this study. The survey was approved by the Bioethical Committee of the Institute of Mother and Child (Opinia nr.3/2018).

## Results

Data were available for 139 women. Table 1 shows characteristics of the women who participated in this pilot study. The participants were in average 29.2±4.7 years of age, the distribution of women in the first, second and third trimester of pregnancy (30.9%, 36.0% and 33.1% respectively) was similar and 42% of the women were in their first pregnancy. Majority of the participants had a normal pre-pregnancy BMI (65.0%) and were never smokers (65.3%). Majority of the women in this study were highly educated (69.1%) and self-classified themselves as having a good or very good socioeconomic status (89.9%). The most common complications reported were vomiting (33/139, 23.7%), vaginal infection (22/139, 15.8%) and anemia (15/139, 10,8%).

**Table 1:** Characteristics of the study sample.

Characteristics of the participants	N = 139	Mean ±SD,[range] or %
Maternal age (years, SD^1^)	139	29.2 ± 4.7 , [18–43]
< 24.9 years	21	15.1%
25–34.9	99	71.2%
≥35	19	13.7%
Gestational age (weeks)	139	21.9 ± 10.8, [5–40]
1^st^ trimester	43	30.9%
2^nd^ trimester	50	36.0%
3^rd^ trimester	46	33.1%
Pre-pregnancy BMI^2^	137	23.2 ± 4.1 , [16.5–36.4]
Underweight	13	9.5%
Normal	89	65.0%
Overweight	33	24.1%
Obese	2	1.4%
Previous pregnancies	138	
0	58	42.0%
≥1	80	58.0%
Smoking status	135	
Never smoker	88	65.2%
Previous smoker	40	29.6%
Current smoker	7	5.2%
Educational level	139	
PhD^3^, Master	71	51.1%
Bachelor	25	18.0%
Secondary	37	26.6%
Low	6	4.3%
Socioeconomic status	138	
Very Good	13	9.4%
Good	94	68.1%
Average	31	22.5%
Marital status	139	
Single	5	3.6%
Married	112	80.6%
Informal relationship	22	15.8%
Planned current pregnancy	138	
Not planned neither expected	22	15.9%
Not planned but expected	22	15.9%
Yes, planned	94	68.1%
Pre-natal education ^4^	50	
Birth school	9	18.0%
Midwife’s school	19	38.0%
None	22	44.0%
Source of information	137	
Reliable	106	77.4%
Not reliable	31	22.6%

1.SD standard deviation

2.BMI body mass index

3.PhD Doctor of Philosophy

4.Pregnant women should be referred to pre-natal education between 21 and 26 weeks of gestation. Women with less than 27 weeks were not included in this analyses.

The pre-pilot questionnaire is available as a supplementary table and had initially 34 questions. Content and face validity evaluation resulted in the modification of 12 response items (lack of relevance of food products, unclear interpretation of responses, wording changes for clearance), removal of five items (questions which were not practical or very difficult) and addition of four extra items. The final questionnaire for nutrition and weight gain knowledge during pregnancy contains a total of 33 items; 26 multiple choice items and seven items with Likert scale (see Table 2). All items had “I do not know” or “I have no opinion” as a possible answer to diminish guessing. The questionnaire comprises four domains: weight gain with 6 items, importance of nutrients with 3, quality of food intake with 13 and quantity of food intake with 11 items.

**Table 2:** Summary of the domains of the questionnaire.

Domains	Description	No. of items
Weight gain	Questions about weight gain during pregnancy	6
Importance of nutrients	Questions about nutrients and vitamins which are necessary or mandatory during pregnancy	3
Quantity of food intake	Questions about portions or servings of foods recommended during pregnancy according to Polish official guidelines	13
Quality of food intake	Questions about foods and drinks recommended or to be avoided according to Polish official guidelines	11

Table 3 shows the results of factor analysis and reliability analysis for all four domains of the questionnaire. All four domains, weight gain, importance of nutrients, quantity and quality of food intake showed KMO>0.5 and Bartlett’s test was statistically significant, indicating appropriateness of factor analysis. Weight gain domain had two dimensions, importance of nutrients one dimension and quantity and quality of food intake domains had three dimensions each. Eigenvalues < 1 were not used in the current construct. The first dimensions of weight gain, quantity and quality of food intake domains had a Cronbach’s alpha > 0.7. Importance of nutrients domain and the second and third dimensions had α < 0.7. Cronbach’s alpha was not calculated for dimensions with one item only. The cumulative explained variance for domains weight gain, importance of nutrient, quantity and quality of food intake was 54.74%, 42.74%, 54.42% and 48.99% respectively.

**Table 3:** Summary of factor analysis and reliability analysis results for all four domains of the questionnaire.

Domains	Dimensions	Number of items	KMO	Eigenvalues	Cummulative explained variance (%)	Cronbach’s alpha
**Weight gain**			0.629			
	1	4		2.111	35.177	0.702
	2	2		1.174	54.737	0.122
**Importance of nutrients**			0.563			
	1	3		1.282	42.742	0.320
**Quantity of food intake**			0.702			
	1	8		3.216	29.235	0.766
	2	2		1.426	42.199	0.490
	3	1		1.344	54.416	-
**Quality of food intake**			0.772			
	1	8		3.323	27.689	0.730
	2	2		1.419	39.516	0.383
	3	2		1.138	48.999	0.067

Footnote: KMO Kaiser-Meyer-Olkin statistics

Tables 4, 5, 6 and 7 describe the items included per domain and the results of factor analysis, reliability analyses, item difficulty and item discrimination for weight gain, importance of nutrients, quantity and quality of food intake domains, respectively. The items loaded varied from 0.442 to 0.830. Alpha deleting item test indicated that removing items one and 21 resulted in an increased Cronbach’s alpha (see Tables 4 and 7). Item 30 did not have load results due to no variability in the answers, that is, all women selected the same answer. Items with less than 40% of correct answers were related to quantity of food intake domain: source of energy, number of daily portions of dairy products, and number servings of fruits and vegetables. Items with more than 90% of correct answers were related to importance of nutrients (for example item “impact of maternal nutrition on the unborn child”) and quality of food intake (for example item “alcohol intake avoidance”). Item13:”should pregnant women eat more fruits or vegetables?” had an ID of 0.77 (item difficulty of 0.91 for the above 75^th^percentile minus item difficulty of 0.14 for bellow 25^th^ percentile). Questions 28 and 40 had higher percentage of correct answers among the bottom 25^th^ percentile scores (ID = -0.18 and ID = -0.03). All women had the same item response for item 37 (ID = 0).

**Table 4:** Weight gain domain: Factor analysis and reliability analysis results.

Weight gain	Item	Factor’s loading	α if item deleted	Item difficulty (%)	Item discrimination
Dimension 1	1.If underweight or little weight gain during pregnancy can be a reason for maternal health problems	0.68	0.74	47.1	0.54
	2.If underweight or little weight gain during pregnancy can be a reason for child’s health problems	0.75	0.67	66.5	0.53
	3. If overweight or excessive weight gain during pregnancy can be a reason for maternal health problems	0.73	0.69	82.5	0.44
	4.If overweight or excessive weight gain during pregnancy can be a reason for child’s health problems	0.74	0.69	65.9	0.58
Dimension 2	5.How many kilos a pregnant women, with similar weight and height to yours, should gain during the entire pregnancy	0.77	-	74.1	0.37
	6.Losing weight during pregnancy is safe	0.54	-	64.2	0.42

**Table 5:** Importance of nutrients domain: Factor analysis and reliability analysis results.

Importance of nutrients	Item	Factor’s loading	α if item deleted	Item difficulty (%)	Item discrimination
Dimension 1	7.Which vitamins and minerals should every pregnant woman take in the form of tablets or capsules^a^	0.67	0.22	44.3	-
	8.What pregnant woman eats has an impact on the health of her unborn child.	0.62	0.27	92.8	0.18
	9.Whether a healthy woman should take folic acid in connection with pregnancy	0.67	0.20	97.1	0.11

a. subitems: vitamin A, vitamin C, vitamin D, zinc, iodine, magnesium, calcium, iron, DHA acid

**Table 6:** Quantity of food intake: Factor analysis and reliability analysis results.

Quantity	Item	Factor’s loading	α if item deleted	Item difficulty (%)	Item discrimination
Dimension 1	10.Whether and when the demand for calories (energy) from food in a healthy pregnant woman increases	0.51	0.75	52.9	0.55
	11.Which type of food should provide the most important source of energy during pregnancy	0.53	0.75	32.1	0.50
	12.What daily portion of dairy products should be consumed by pregnant women	0.74	0.72	2.9	0.09
	13.Should a pregnant woman eat more fruits or vegetables	0.53	0.76	50.0	0.77
	14.How many servings of fruits a pregnant woman should eat daily	0.83	0.68	36.2	0.14
	15.How many servings of vegetables a pregnant woman should eat daily	0.68	0.72	33.3	0.57
	16. How portions of fruits and vegetables should be spread out during the day	0.56	0.75	68.4	0.53
	17.How many times a week a pregnant woman is recommended to eat fish	0.42	0.77	58.3	0.37
Dimension 2	18.How many meals a day should be eaten during pregnancy	0.60	-	80.1	0.11
	19.How much liquids a pregnant woman should drink per day	0.69	-	84.8	0.23
Dimension 3	20.Should a pregnant woman eat for two	0.66	**-**	89.9	-0.18

**Table 7:** Quality of food intake domain: Factor analysis and reliability analysis results r.

Quality	Item	Factor’s loading	α if item deleted	Item Difficulty (%)	Item discrimination
Dimension 1	21.Which cereal products should a pregnant woman choose	0.55	0.75	84.8	0.40
	22.Which products a pregnant woman should not eat^a^	0.71	0.69	70.5	-
	23.What kind of snacks are recommended between meals^b^	0.66	0.69	85.6	-
	24.What kind of drinks are recommended for a pregnant woman^c^	0.42	0.72	89.6	-
	25.Which fat products are recommended during pregnancy^d^	0.71	0.68	44.2	-
	26.Which products contain unhealthy fats which are contraindicated during pregnancy^e^	0.63	0.70	61.1	-
	27.Which products are a good source of protein in the diet of pregnant women^f^	0.62	0.69	65.1	-
	28.Which fish are not recommended during pregnancy^g^	0.68	0.68	40.9	-
Dimension 2	29.If and which portion of strong alcohol is harmful during pregnancy	-	-	100	0
	30.If and which portion of beer is harmful during pregnancy	0.57	-	96.4	0.09
	31.If and which portion of wine is harmful during pregnancy	0.75	-	91.3	0.20
Dimension 3	32.If and which portion of coffee is harmful during pregnancy	0.68	**-**	96.4	-0.03
	33.What type of meat and meat products are recommended during pregnancy	0.55		54.2	0.31

**a.** Subitmes: raw unpasteurized milk, milk UHT, blue cheese, cottage cheese, feta cheese, boiled eggs, raw smoked meat, roast beef, tatar, liver, sushi with raw fish, leguminous, nuts, sprouts and packed salads, kogel-mogel

**b.** vegetables, potato chips, almonds, pumpkin or sunflower seeds, salted nuts, fruit jelly, cakes and chocolate bars

**c**. still water, sparkling water, flavored water, soft/soda drinks, low fat milk, energy drinks, fruit juice, vegetable juice

**d.** butter, soft margarine, hard margarine for baking, olive oil, rapeseed oil, coconut oil, lard

**e.** cakes and chocolate bars, nuts such as peanuts or pistachio, salty snacks such as crackers and sticks, sauces and powdered soups, kabanos sausage

**f.** milk and milk products, meat, fish, groats and rice, cruciferous vegetables, legume

**g**. carp, salmon, panga, trout, herring, tilapia, smoked mackerel.

## Discussion

The purpose of this study was to test the adequacy of the 33-item questionnaire as a research instrument. The results of this study indicate that the questionnaire is stable and internal consistency is acceptable (Cronbach’s alpha > 0.7) for dimensions with more than four items. For dimensions with less than four items, internal consistency was poor (Cronbach’s alpha <0.7). These dimensions were kept in the questionnaire concept due to their relevant content.

There are only a few studies available in the literature assessing the level of nutritional and weight gain knowledge during pregnancy. For the few studies available, some are missing measures of validity and reliability and some questionnaires are not based on updated recommendations [42].

The level of nutrition, awareness of weight gain and diet adequacy to official guidelines was explored in a group of pregnant women from Australia [25,43,44]. The domains reported by the authors appear to be similar to the domains of our study regarding importance of nutrients, knowledge on weight gain and quality of food intake. Estimates of validity and reliability of the questionnaire are described and appears to be sufficient. The main difference between this questionnaire and ours is that the Australian questionnaire also measures attitudes toward key nutrition topics and in our questionnaire, we focused specifically on the level of knowledge.

Other studies explored the same topic, but in a segregated fashion. One study with pregnant women from Romania examined nutritional knowledge as a determinant of mineral supplementation [45]. The authors used a standardized questionnaire which evaluated nutritional recommendations and sources of nutrients. The Romanian questionnaire contains some of the items included in our study as well, however without the organization within domains. The Romanian questionnaire has diet-related disease items and our questionnaire focuses on ensuring whether or not pregnant women know they need to take certain essential minerals and vitamins, even though they might not know the specific disease related to it.

In another study, a group of Australian pregnant women was asked to indicate in a survey whether certain groups of foods were safe, should be avoided or limited to eat during pregnancy [46]. In our study, in the quality domain, participants were asked which food should not be eaten by pregnant women, focusing on safety of food consumption. Food groups associated with a risk of foodborne illness such as listeriosis or toxoplasmosis, associated with a risk of high mercury exposure or miscarriage or complications or birth defects were assessed either directly named, for example raw meat or raw fish, or with the name of the meal containing raw meat or fish; tatar or sushi or “*kogel-mogel*”, a regional dish with raw eggs.

None of the studies cited above addressed in depths the knowledge of quantity of food intake during pregnancy. According to item difficulty analysis in our study, the knowledge of women regarding caloric intake and number of daily portions of food needs substantial improvement.

The first domain of this questionnaire, weight gain knowledge, has also been assessed separately in other studies. Most studies contains at least the evaluation whether women follow the guidelines to proper weight gain according to their respective weight and height [47–49]. In addition, our questionnaire contains items asking pregnant women whether they know that little or excessive weight gain has an impact on maternal and child’s health.

Correct answers in this questionnaire are based on official international guidelines for healthy eating during pregnancy [38] which is relatively similar throughout countries and should only differ by regional specifications, depending on the malnutrition profile of the region. High income countries have a western diet pattern while low and middle income countries are transitioning from a traditional dietary pattern to a western pattern [50] and maybe also be dealing with inequality of food access or food shortages. In both situations, the double burden of malnutrition, i.e., the simultaneous presence of undernutrition and obesity, might be observed [51,52]. However some countries might have certain popular food items which affect the health of the mother or the child. For example, in some countries in Asia or South America dealing with inequality of food access or food shortage, palm leaves is a possible source of protein [53]. In this study, in the domain “quality of food intake”, kogel-mogel (raw eggs) and kabanos sausage (pork processed meat) were added as answer options. Therefore, some adaptations in the questionnaire due to prevalence of certain local dishes may be necessary. Nevertheless, these adaptations are not structural and might not invalidate the generalizability of the questionnaire, given that the vast majority of the items are based on international recommendations for healthy eating during pregnancy. Moreover, the domains weight gain, importance of nutrients, quantity, and quality of food intake in this questionnaire should be capable of detecting suboptimal knowledge associated with undernutrion as well as overnutrition.

One limitation of our study is the poor reliability of dimensions with less than four items. The poor reliability might be related to the limitation of Cronbach’s alpha analysis in providing true reliability when dimensions have fewer items.

Strengths of this study include the development of a questionnaire which is adapted to the eating habits and cultural specifications of the Polish population. The source population in which this questionnaire was evaluated is going through a nutritional transition towards westernization of the diet [52]. Poland had a state controlled economy and the population was less exposed to fast food or ultra-processed foods and traditional cooking is still quite popular [53]. Therefore, the questionnaire included items with regional dishes which should be avoided or are recommended to pregnant women. Additionally, the questionnaire was developed with the aid of nutritional experts, who ensured all aspects of nutrition during pregnancy were assessed.

## Conclusion

Pregnancy is a period in life in which women are willing to change their lifestyle. Providing precise information for these women is crucial for their health and the health of their offspring. To indicate weak points on their nutritional and weight gain knowledge is the first step to improve nutritional health care assistance. The proposed questionnaire has shown to be a proper instrument for the purpose it was designed for.
